# Some Account of the Work of Dr. Michaels

**Published:** 1902-04

**Authors:** Edward C. Kirk

**Affiliations:** Philadelphia


### International Dental Journal.

VOL. XXIII. APRIL, 1902. No. 4.

### Original Communications.[1]

### SOME ACCOUNT OF THE WORK OF DR. MICHAELS.[2]

BY EDWARD C. KIRK, D.D.S., PHILADELPHIA.

WHILE in conversation with Dr. Truman to-day the thought was evolved that the members of the Academy might be interested in learning something about the work that Dr. Michaels has been carrying on for the past six years. Last year a paper was published in the INTERNATIONAL DENTAL JOURNAL, in which Dr. Michaels gave his views of the relation of the saliva to the production of erosion or chemical abrasion of the teeth, and made the remarkable statement that it was due to sulphocyanide of potassium. I had the opportunity a year ago of listening to his paper before the International Congress in Paris, and afterwards made a rather careful study of the paper upon sialo-semeiology, and I made up my mind that if the opportunity afforded I would take occasion to make personal investigation of his remarkable claims, for they are remarkable. Hitherto the saliva has been regarded simply as a fluid containing a ferment, ptyalin, capable of convert-

[1] The editor and publishers are not responsible for the views of authors of papers published in this department, nor for any claim to novelty, or otherwise, that may be made by them. No papers will be received for this department that have appeared in any other journal published in the country.

[2] Remarks made before the Academy of Stomatology, October 22, 1901.

ing starch into maltose, also some salts, mucin, and water. That is to say, it is a fluid which has a certain digestive property, but is mainly useful for lubricating a bolus of food so that it may be comfortably swallowed. Dr. Michaels, as the result of his studies, takes an entirely different view of the question. Apart from its physiological function, he regards the saliva as affording the very best medium which we have for arriving at a conception of the status of nutrition. He says the saliva more accurately represents the composition of the blood than any of the other bodily fluids. Physicians have been investigating the urine with reference to normal nutrition and pathological conditions, but Dr. Michaels regards the urine as of secondary importance in this relation. He says that it is an excretion by a gland specialized in character, and does not stand in such close physiological relationship to the blood-current as does the saliva. He holds that the saliva represents the blood-current more accurately, because it is a secretion from the blood, and that it may contain everything in the blood-stream which is crystallizable and therefore that is dialyzable through a glandular structure. He says that the saliva is swallowed as it is secreted and passes through the nutrition-cycle over and over again, and in performing this evolution it picks up the soluble materials in the blood-stream and presents them in solution.

I became interested in the matter, because, in the study of the diseases of the mouth, the theories held with reference to caries, and the question of erosion of the teeth, as well as of the invasion of the soft tissues of the retentive apparatus by bacteria, it seemed to me that the whole atmosphere of dental pathology had become so beclouded with the bacteriological question that we could not see beyond it. We had gotten to the point where we were regarding every disease as produced by bacteriological influence, and had lost sight of the important fact that there were many cases in which, though bacteria were present, the same class of organisms that were exciters of diseased conditions, yet no pathological results occurred. There is something in the nutritional status of the individual, and that nutritional status being, of course, related to and dependent upon conversion of food in the organism, through the process of cell metabolism, into energy, tissue, and waste products, by which process there is produced somewhere along the line of nutrition the state which we call vitality or life. The question of internal vital resistance to disease invasion seems to be involved in the process of nutrition, and has a most important bearing on the predisposing causes of disease.

Dr. Michaels has found, and I think he has very clearly demonstrated, that the percentage and character of salts found in the saliva bear a distinct relation to the intensity of the disease. As the blood-current becomes loaded up with these crystallizable waste products of nutrition, there is an osmotic overflow into the saliva; therefore, the quantity of these contained salts is important evidence of the state of the blood, which in turn is dependent upon the nutritional status. He claims to be able to determine from an examination of the saliva and blood the disease or diathetic state. It seems from his researches that it is an extremely simple matter to be able to differentiate individuals into two large classes,—those whom he calls the "hyperacid" individuals, whose saliva contains acid calcium and sodium phosphates, and possibly urea or uric acid salts, in quantities which may be detected and determined by the microscope. These salts exist in what he calls the "hyperacid diathesis," of which the rheumatic and gouty individuals are well-known examples. On the other hand, there is an opposite type which he calls the "hypo-acid individual," whose condition is characterized by a different class of salts in the saliva. After a little practice one may, with great ease and readiness, recognize the two classes of salts, and so determine the diathetic states.

The technique of examination is extremely simple. A specimen of mixed saliva of all the glands is taken. The mouth is first rinsed with water, so as to remove the food particles; no attempt at sterilization is made. After a sufficient quantity—a dram or two—is collected, then, with a dropping-tube several drops are deposited on a white porcelain tablet and the saliva is rapidly put through a series of qualitative tests, in order to determine its general character. The first test is made with one of the ferric salts, usually ferric chloride, in ten per cent. solution, in which we get a reaction showing the sulphocyanide of potassium, if it be present. The intensity of the color determines, to a certain degree, the diathetic state. Dr. Michaels has found in the gouty individual of the hyperacid diathesis that the sulphocyanide tends to disappear, so if he finds a saliva not giving any or a weak reaction to ferric chloride, he begins to suspect that the individual is of a gouty type. If the individual is of a rheumatic character and the amount of sulphocyanide is above normal, you get a more vivid coloration. When we speak of being "above" or "below" normal, the normal standard is arrived at by making examinations of saliva with respect to the sulphocyanide test in a number of individuals who are in normal health. So you obtain after a while a conception of the standard of coloration, and you can determine whether the coloration is too intense or wanting in intensity.

Another test that is made on the porcelain tablet is for the existence of ammonia or ammoniacal salts, and is done by Nessler's reaction, which gives a characteristic brownish color if any ammoniacal salts are present. They may or may not be present. Chlorides are determined by the addition of silver nitrate in a decinormal solution, using yellow potassium chromate as an indicator, which is a well-known test for chlorides in the laboratory. He makes a test for glycogen, the presence of which he regards as a measure of health in the individual. If it be present in the saliva, he says that individual is in good health. It is the material into which the proteid substances taken in as food are converted in an available form for assimilation, and its presence in the saliva indicates that the system has stored up a quantity more than sufficient for its needs. Its absence does not mean ill-health, but its presence is an indicator which has a strong bearing on the existing status of the nutrition.

The third test on the porcelain tablet is the reaction of the saliva with reference to litmus paper. A great deal of light has been thrown on the question of salivary reaction by the work of Michaels. It is put down in the text-books as being alkaline or neutral. It may be strongly acid, alkaline, or neutral, or may be both alkaline and acid,—*i.e.*, amphoteric. That is to say, where the saliva contains disodium phosphate, and where it also contains the acid sodium phosphate or acid calcium phosphate, one may get in the same saliva a reaction to either blue or red litmus paper. The same condition is found in urine. So the acidity or alkalinity is to be expressed in terms of the proportions of sodium phosphate or acid calcium phosphate present.

Dr. Michaels lays a great deal of stress upon the mode by which various kinds of saliva undergo decomposition. I saw salivas of all ages that he has kept for weeks, months, and even years, and these salivas were decomposed in various ways. Salivas from patients affected with some derangement of the liver decompose with a dark brownish or greenish coloration. In the hyperacid cases, the rheumatic and diabetic cases, the color will be more of a reddish type, and these color results of decomposition form part of the history of the case which enables him to make up his diagnosis.

The most striking feature of his investigation is the microscopical study of the salts in the saliva after they have been crystallized. In all the works upon urinalysis and physiological chemistry that I have studied—and I have gone over most of the standard authors, both in English and in French—I have found no reference but one to the use of the polariscope in the study of these crystalline forms; though the study of the urine sediments and crystal forms is now elaborately worked out. Dr. Michaels has laid great stress upon the use of the polariscope in the study of these crystalline forms in the saliva. He differentiates those salts which are polarizable from those which are not, and thus makes a subclassification. He has found all the acid salts to be highly polarizable. The whole process seems to resolve itself into two things,—first, the study of crystalline forms in saliva, with a view to understanding its chemical composition; that is, by making an analysis of it by the microscope and chemically by the use of reagents. Secondly, to understand the pathological significance of these findings, whatever they may be. What does it mean when we find so much urea and acid calcium phosphates in the saliva? The bearing of such questions on diseased conditions is the final and most important problem.

I went so far with Dr. Michaels as to study his general methods, and it seems to me that I have got to a point where I am ready to begin the work. I have spent some months on it now. By beginning the work I mean seriously to take up the systematic study of salivas of a uniform line of cases of the same disease and to see if there is any common factor running through them, and to study those conditions as compared with normal conditions.

Dr. Michaels asserts positively that he is able to make a diagnosis of any well-known disorder by his method. The technique of making specimens is quite simple. He makes them in duplicate. A large drop of the saliva is deposited on a microscopical slide and covered with a round cover-glass. Alongside of that is placed an uncovered drop of saliva. One such slide is placed in a warm chamber so that evaporation takes place at about 105° F. That gives him specimens for immediate study. The other is allowed to dry spontaneously at room temperature, being merely covered over with a bell-glass to prevent particles of dust from coming into it, and is then studied under a microscope. I have brought with me to-night the microscope with a polariscopic attachment and some specimens which I should like to show to as many as can see them. I think you will agree that the appearances shown in these specimens of saliva taken from diseased individuals are, if not characteristic, at least strongly suggestive of that idea, and hold out the promise of great utility in the matter of diagnosis. Bear in mind that the quantity of salts present seems to be related to the intensity of the disease, and not only enables us to say, "This man is of such a diathesis," but when the characteristic salts are present in small quantities we will not regard him as a very sick man, but where the saliva is loaded down with them we have reason to suspect that the man is very ill; in the cases that I have studied thus far this fact seems to be borne out. If this investigation yields many of the results which it promises, I believe that we have one of the most important means of diagnosis given to us in recent years. It furnishes us with a key that, after awhile, may present the problem of caries from an entirely different view-point. The question is constantly being asked why it is that this or that particular theory does not fit a certain case. I have very great doubts as to whether the type of caries seen in the mouths of young children or adults, where approximal surfaces are attacked and where the disease is running rampant, is the same as that found in the gouty or hyperacid type of individual, where it seems that an acid substance is exuded from the gum margin and where decalcification is girdling the tooth. Dr. Michaels finds that these different types of caries are related to diathetic states and to variations in the composition of the oral fluids, and that they have different causes. The nearer we get to an understanding of the original causes of this disease the better able will we be to prevent and cure it. I must say, with reference to my study with Dr. Michaels, that it was exceedingly gratifying and was one of the most hopeful things that I have met in dental pathology.

